# The importance of temporal-fine structure to perceive time-compressed speech with and without the restoration of the syllabic rhythm

**DOI:** 10.1038/s41598-023-29755-x

**Published:** 2023-02-18

**Authors:** Robin Gransier, Sara Peeters, Jan Wouters

**Affiliations:** Department of Neurosciences, ExpORL, KU Leuven, Herestraat 49, Box 721, 3000 Leuven, Belgium

**Keywords:** Auditory system, Sensory processing, Neuroscience, Perception

## Abstract

Intelligibility of time-compressed (TC) speech decreases with increasing speech rate. However, intelligibility can be restored by ‘repackaging’ the TC speech by inserting silences between the syllables so that the original ‘rhythm’ is restored. Although restoration of the speech rhythm affects solely the temporal envelope, it is unclear to which extent repackaging also affects the perception of the temporal-fine structure (TFS). Here we investigate to which extent TFS contributes to the perception of TC and repackaged TC speech in quiet. Intelligibility of TC sentences with a speech rate of 15.6 syllables per second (sps) and the repackaged sentences, by adding 100 ms of silence between the syllables of the TC speech (i.e., a speech rate of 6.1 sps), was assessed for three TFS conditions: the original TFS and the TFS conveyed by an 8- and 16-channel noise vocoder. An overall positive effect on intelligibility of both the repackaging process and of the amount of TFS available to the listener was observed. Furthermore, the benefit associated with the repackaging TC speech depended on the amount of TFS available. The results show TFS contributes significantly to the perception of fast speech even when the overall rhythm/envelope of TC speech is restored.

## Introduction

Speech perception is facilitated by the integration of different speech segments over time. Running speech can be characterized by two acoustic temporal features, namely the temporal envelope (E; also referred to as the amplitude envelope) and the temporal fine structure (TFS)^1^. Whereas the former provides information about the timing and intensity of the articulated speech segments, the latter provides information about temporal changes of the place and manner of the articulated speech segments (i.e., the spectral changes over time)^2^. Although normal-hearing listeners can perceive speech in quiet based on either the E or the TFS, speech perception in quiet based on the E is highly robust^3–6^. Furthermore, the E is used in cochlear implants to enable speech perception for the severely-hearing impaired^7^. However, speech perception based purely on the E is insufficient when listening conditions become challenging, e.g., when speech is presented in noise or when a competing talker is present. In these cases, access to the TFS, in addition to the E features benefits speech perception^8–12^.

Most studies that investigate the contribution of the E and TFS to speech perception leave the timing of the individual speech segments intact. However, the importance of the timing of the individual speech segments is clear from studies that manipulate E, for example by increasing the speech rate^13–15^. Time compression, in which the speech rate is increased, but the TFS is kept intact^16^, is often used when investigating the upper limit of temporal processing for speech perception^17–19^. Although the upper limit of young normal-hearing listeners to perceive 50% of the time-compressed (TC) speech ranges from 11 to 16 syllables per second (sps)^17–19^, it is also characterized by a large inter-subject variability^17,20^. Furthermore, a reduced ability to perceive TC speech—not related to any peripheral hearing deficits—is associated with advancing age^19,21,22^ and central auditory processing deficits^23^.

It has been hypothesized that the ability to perceive fast speech is limited by the cortical processing mechanisms^14,24,25^. Ghitza and Greenberg^14^ postulated that the decline in intelligibility with increasing speech rate is the result of a disruption in the syllabic rhythm beyond the limits of what the cortical processing mechanisms can handle. This hypothesis is in line with the spectro-temporal excitation pattern (STEP) model of Moore^26^, which assumes that the cortex maps incoming sounds with its internal representations. In case the incoming speech signal violates these internal representations, intelligibility is affected. Interestingly, Ghitza and Greenberg^14^ found that the intelligibility of TC speech can be significantly improved when silences are inserted between the TC speech segments so that the syllabic rate is restored to normal. In the conceptual TEMPO framework^14,24,27^, Ghitza^24^ hypothesized that this ‘repackaging’ process enables an optimal match between the spectro-temporal modulations and a cascade of neural oscillators that parse and decode the different E and TFS of the speech signal. In the TEMPO framework, a theta oscillator (4–10 Hz) that synchronizes to the E, is assumed to drive a cascade of neural beta (16–40 Hz) and gamma (> 60 Hz) oscillators which in turn parse and decode the speech signal at a more fine-grained level (i.e., the TFS). Several studies have found that when the E modulations of TC speech exceed the operating range of the theta oscillator, speech perception deteriorates^17–19,25^. These findings are in line with electrophysiological studies that found that the auditory cortex is more efficient in processing low-frequency temporal fluctuations compared to high-frequency temporal fluctuations^28–30^. In addition, Ahissar et al.^15^ reported that the ability of the auditory cortex to phase lock to the E of speech was reduced or absent in people with dyslexia who were not able to perceive speech presented at fast rates. Less is, however, known about the importance of the TFS on the intelligibility of TC speech and how it contributes to the intelligibility of repackaged TC speech.

One could hypothesize, within the scope of the TEMPO framework, that if the theta oscillator is not perfectly in sync with the E, the beta and gamma oscillators, that operate -although not optimally synchronized- at a much higher sampling frequency are still able to decode parts of the TFS. As a result, intelligibility of TC speech would significantly be impacted when the access to the TFS is limited, as is the case for vocoded speech or speech transmitted by cochlear implants^18^. Furthermore, this hypothesis suggests that when the theta oscillator is in sync with E, the cascade of oscillators is optimally synchronized to parse and decode the TFS, and hence intelligibility of (repackaged) TC speech depends both on the E and TFS content in the speech signal. Here we investigated this hypothesis by assessing to which extent TFS affects the ability to perceive TC and repackaged TC speech. More specifically, we assessed the effect of a reduced spectral resolution on the ability of young normal-hearing adults to perceive TC and repackaged TC speech.

## Methods

### Participants

Fourteen young adults (mean age = 22.3 years, SD = 1.5 years, range = 20–25 years, male = 4) participated in the study. All participants reported normal hearing and had hearing thresholds ≤ 20 dB HL for octave frequencies ranging from 250 to 8000 Hz. Except for one subject who’s left-ear’s hearing threshold at 250 Hz was 35 dB HL. The Ethics Committee of the UZ Leuven (University Hospital Leuven) approved this study (approval number: B322201941114). The research was performed in accordance with the applicable guidelines/regulations for testing on human subjects and in accordance with the Declaration of Helsinki. An informed consent was obtained from every participant before participating in the study.

### Overview, stimuli, and procedure

Noise vocoders were used to assess to which extent the availability of TFS in the speech signal affects the ability to perceive TC and repackaged TC speech. The intelligibility of the TC sentences and repackaged TC sentences was assessed for three different spectral conditions, namely the original spectral content and the reduction of the spectral content with either a 16-channel or an 8-channel noise vocoder. The average speech rate to assess the effect of TFS on intelligibility was 15.6 syllables per second (sps) for the TC speech, whereas the repackaged TC speech had an average speech rate of 6.1 sps. In addition, in two control conditions we also assessed the intelligibility of the original speech rate (2.5 sps) and that of TC speech at a rate of 6.6 sps. These conditions were added as it is expected that all listeners were able to obtain a 100% intelligibility for these conditions^17^. Furthermore, test–retest reliability was assessed for all conditions that contained TC speech (i.e., spectral content, time-compression, and repackaging).

The speech corpus of the Leuven Intelligibility Sentences Test (LIST)^31^ was used. The LIST corpus is specially designed to precisely assess speech perception in cochlear-implant users and therefore has an average syllabic rate of approximately 2.5 sps. It consists of 350 sentences across 35 lists of 10 sentences and each list has 32 or 33 keywords that are used to score the keywords correctly understood. The LIST corpus was chosen since the sentences are comparable to those used in everyday encountered speech, it has an average number of 9 (SD = 2.2, range = 4–15) syllables per sentence, and the occurrence of the different phonemes in the corpus is comparable to that of Dutch speech^31^. Furthermore, the LIST sentences are clear speech and thereby limit the effects of coarticulation on speech intelligibility^32^. An advantage of clear speech for this experiment is that it enables a better segmentation of the TC sentences into individual syllables. This in turn prevents potential artificial distortions that might have occurred during the segmentation and repackaging process. Some examples of the LIST corpus are (English translation between brackets): “Het vliegtuig stortte neer” (“The airplane crashed”), “Er is genoeg plaats op de bank” (“There is enough space on the couch”), and “Aardbeien zijn zomervruchten” (“Strawberries are summer fruits”).

Sentences were TC at two compression ratios, namely 0.4 and 0.166. The former compression ratio was chosen, since we previously found that this is—by approximation—the highest compression rate where listeners are still able to obtain 100% intelligibility in quiet^17^. Furthermore, the corresponding syllabic rate (i.e., 6.6 sps) is similar to the syllabic rate that has been reported to result in the largest intelligibility benefit when (fast) TC sentences were repackaged, by adding silences, to this rate^24,25^. This compression rate was therefore used as a control condition in the experiments, as it was expected that all listeners would obtain a 100% intelligibility. The latter compression ratio was used in the main experiment and resulted in an average syllabic rate of 15.6 sps. This compression ratio was chosen as it corresponds to the average speech reception threshold (i.e., the compression ratio that results in a 50% correctly perceived keywords) when exactly the same speech corpus was assessed in a group of nineteen young adults with normal hearing^17^. We hypothesized that a compression ratio that resulted in an average intelligibility score of 50% would be optimal to assess whether the addition of silences would improve speech perception, while at the same time prevent a floor effect from happening in case of a potential degrading effect of spectral content on intelligibility. This compression ratio was therefore assumed to reduce the chance of any floor or ceiling effects from occurring in any of the conditions. Furthermore, the average syllabic rate after repackaging the syllables by inserting 100 ms silences resulted in an average syllabic rate of 6.1 sps. This rate is similar to the syllabic rate of the control condition and to that of the repackaged syllabic rate reported in the literature that used the same experimental paradigm^14,25,33^. For more details about the effect of the time-compression on the duration and the rate of the different speech segments, we refer to Gransier et al.^17^; i.e., the study where the TC version of the LIST corpus was first introduced and where the characteristics of the different speech segments as a function of compression ratio are described in detail.

TC speech was generated with the Pitch Synchronous Overlap and Add algorithm^34^, as implemented in PRAAT^35^. The Pitch Synchronous Overlap and Add algorithm was used as it shifts the modulation spectrum with increasing compression ratio to higher modulation frequencies, in contrast to non-uniform time-compression algorithms, but at the same time preserves the shape of modulation spectrum and the spectral features of the speech signal^16^.

**Figure 1:**
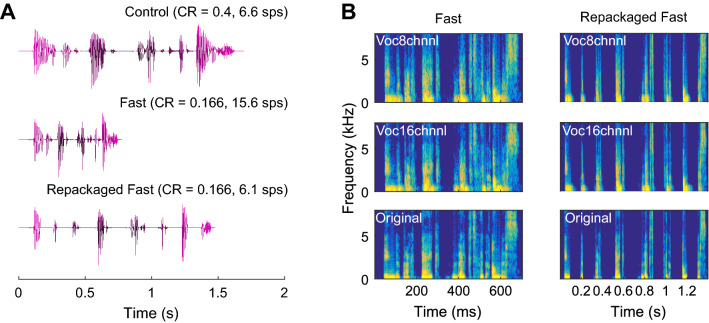
(**A**) The time waveforms of the sentence “in de praktijk werkt het anders” (“in practice it works differently”) when TC with a compression rate (CR) of 0.4 (i.e., control condition) and 0.166 (i.e., fast condition), and the repackaged fast condition by adding 100 ms of silence between the syllables. (**B**) The spectrograms, of the same sentence as in (**A**), of the different TFS content for both the fast (left) and repackaged fast (right) sentences.

The repackaging of the TC speech with the added silences was done in PRAAT ^35^. First, each TC sentence was manually segmented in individual syllables. Segmentation was done at zero-endpoints to prevent the introduction of artificial transients in the repackaged speech. Thereafter, 100 ms of silence was added after each syllable and all silence-included syllables were concatenated in the same order as the original speech. Figure 1A shows the waveforms of the control, the fast, and the repackaged fast sentence.

Sentences were vocoded using the same procedure as in Van Hirtum et al.^36,37^ and was done in MATLAB R2016B^38^. Each sentence was first filtered in either eight (8-channel vocoder) or sixteen TFS channels (16-channel vocoder) using an analytical filter bank based on fourth-order Butterworth filters. The cutoff frequencies for the 8-channel vocoder were: 187.5, 437.5, 687.5, 1062.5, 1562.5, 2312.5, 3437.5, 5187.5, and 7937.5 Hz, whereas those for the 16-channel vocoder were: 187.5, 312.5, 437.5, 562.5, 812.5, 1062.5, 1312.5, 1562.5, 1812.5, 2187.5, 2687.5, 3187.5, 3812.5, 4562.5, 5437.5, 6562.5, and 7937.5 Hz. Second, the TFS channels were half-wave rectified and filtered with a fourth-order Butterworth filter, with a cutoff frequency of 200 Hz, to extract the envelope of each channel. Third, individual noise channels were created by filtering a broadband noise carrier with the same filter characteristics as used to obtain the TFS channels of the speech signal. Individual noise channels were then modulated with the envelope of the corresponding TFS channel. Finally, the envelope-modulated noise channels were matched in level to their original in-band input and summed to create the vocoded speech signal (see Fig. 1B for the spectrograms of the different conditions).

In order to have enough speech material and at the same time to reduce the time needed to manually segment each of the TC sentences, which was needed to add the 100 ms silences to assess the restoration effect, we first randomly selected six lists from the LIST corpus (i.e., 6 × 10 sentences, selected lists: 10, 13, 18, 23, 30, and 33). These sentences were TC at the fastest rate, then manually segmented, and the 100 ms silences were added between the segments. Second, nine lists, different to the six already selected lists, were then randomly selected from the LIST corpus (i.e., 9 × 10 sentences, selected lists: 6, 8, 9, 16, 21, 22, 25, 28, and 35). These nine lists were used to assess the intelligibility in the conditions in which no silence was added. Third, we used the one list of the LIST corpus that was not selected during the first two steps as a back-up list. This list was used in case any of the conditions needed to be reassessed due to e.g., technical issues. This only happened once in the whole study. This selected speech corpus was large enough to assess each condition with an unique list in each participant.

Prior to each experiment (i.e., per participant) we randomly assigned each list from the preselected corpus, as described above, to each condition. For example, for one participant, the list used to obtain the intelligibility scores for each of the six conditions that assessed the effect of spectral content on the improvement in intelligibility, when the 100 ms silent gaps were added between the syllables of the TC sentences, were 13, 33, 10, 18, 30, and 23, for respectively the condition 0.166 CR 8-Channel Vocoded Test, 0.166 CR 16-Channel Vocoded Test, 0.166 CR Original Test, 0.166 CR 8-Channel Vocoded Retest, 0.166 CR 16-Channel Vocoded retest, and 0.166 CR Original retest. Whereas for another participant the list 18, 30, 23, 10, 13, and 6, was used to assess these conditions, respectively. As a result, each condition was assessed with an unique list, and the list used to assess the different conditions differed across subjects. This approach was chosen so that the results were not biased by a specific combination of list (i.e., specific sentences) and condition (e.g., number of channels * speech rate) on the overall results. The expected bias, however, is assumed to be negligible given that the sentences were drawn from a normalized corpus of sentences equal in spectral and temporal content^31^.

Table 1 shows the different conditions that were assessed in a single 1.5- to 2-h session. The original-speech rate and original-spectral content condition was included since normal-hearing listeners easily achieve 100% intelligibility when listening to these sentences in quiet ^31^. Therefore, any subject that would not be able to achieve a 100% intelligibility was assumed to have suprathreshold hearing deficits and would therefore be excluded in the analyses. However, this was not the case in the present study. Conditions (i.e., a single condition was assessed with one list) were assessed in two separate blocks and in between blocks there was a small break. In the first block, all conditions were presented in pseudo-random order. We refer in the following to the data collected in the first block as the test data. In the second block, all the TC conditions were assessed for a second time but in a different pseudo-random order, we refer to this data in the following as the retest data. By assessing speech intelligibility for the TC conditions twice (i.e., test and retest) we were able to assess whether short-term familiarity with the speech material affected the intelligibility of the TC and repackaged TC sentences. For both the test and retest, the pseudo-randomization of the conditions differed across participants so that no effect of presentation order could affect the results.

**Table 1:** The different conditions assessed during the experiment.

Speech rate	Spectral content	Silence added
Original	Original	No
0.4 CR^a^	Original	No
0.166 CR^a^	8-channel vocoder	No
0.166 CR^a^	16-channel vocoder	No
0.166 CR^a^	Original	No
0.166 CR^a^	8-channel vocoder	Yes
0.166 CR^a^	16-channel vocoder	Yes
0.166 CR^a^	Original	Yes

Each condition was assessed with a single list consisting of ten sentences.

^a^Condition was assessed both in the test and retest assessment.

All conditions were presented in half of the study group to the left ear, whereas in the other half of the study group all conditions were presented to the right ear. Which ear was tested per participant was pseudo-randomly determined. This was done to exclude any potential bias of ear of stimulation.

After hearing each sentence the participants were asked to repeat aloud what they had heard. The intelligibility score per list was based on the number keywords correctly recalled.

The Apex experimental platform^39^, interfacing with a RME Fireface UC, and connected to Sennheiser HDA200 headphones was used to present the speech material to the participant. The speech sentences, in all conditions, were presented at an A-weighted sound pressure level of 65 dB re 20 µPa. The sound emitting system was calibrated with a sound level meter [Brüel and Kjær (B&K)-type 2250] connected to a ½ in. microphone (B&K—type 4192) which was placed in an artificial ear (B&K—type 4153).

### Statistical analysis

All statistical tests were carried out in R (version 4.1.1)^40^. In the following we report speech intelligibility as the percentage keywords correctly recalled. For the statistical analysis of normality and the post-hoc tests the percentage keywords correctly recalled were transformed to rationalized arcsine units (RAU) ^41^. Normality of the data was confirmed by Q–Q plots and Shapiro–Wilk tests, and the homogeneity by means of residual plots.

A t-test was used to assess the effect of speech rate on the intelligibility between the control condition (syllabic rate = 6.6 sps) and the speech rate used in the main experiment (syllable rate = 15.6 sps).

A repeated-measures ANOVA with the percentage keywords correct as dependent variable, subject as between variable, and adding silences, spectral content, and measurement (test/retest) as within variables was used to assess the effect of amount of TFS available to the listener and the repackaging effect on the intelligibility of TC speech. Greenhouse–Geisser correction was used in case the Mauchly’s test indicated that the assumption of sphericity had been violated. Pairwise-T tests based on the RAU scores were used in the post-hoc comparisons. The post-hoc analysis assessing the interaction between the effect of adding silences and spectral content was based on the benefit that listeners experience in the repackaged TC speech conditions compared the TC speech conditions.

A significance level of 5% was used in all analyses and a Bonferroni correction was applied for the post-hoc comparisons.

## Results

All participants obtained 100% intelligibility for the sentences with the original spectral content and the original speech rate.

In the control condition, which consisted of TC speech with an average syllabic rate of 6.6 sps, participants obtained an average intelligibility across the test and retest condition of 99.8% (SD = 0.8%, range = 96.9–100%). However, the average intelligibility across listeners was significantly reduced [*t*(28.794) = −21.647, *p* < 0.00001] to 41.9% (SD = 18.0%, range = 12.5–84.4%) when the time-compression ratio increased to an average rate of 15.6 sps.

**Figure 2:**
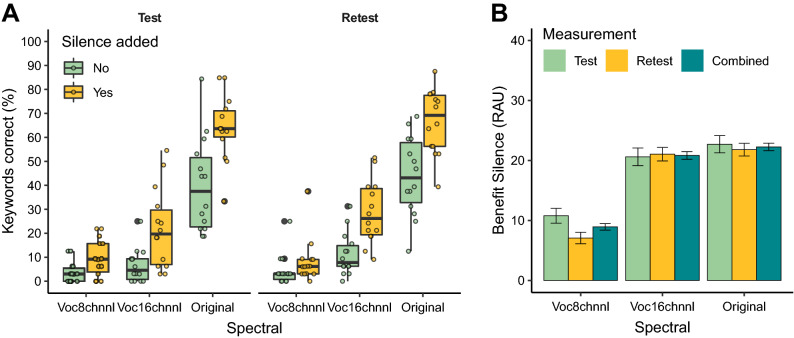
(**A**) Effect of spectral content, addition of silences, and measurement (test and retest) on the percentage of correct recalled keywords. (**B**) The benefit (based on the RAU scores) on intelligibility when adding the 100 ms silence segments between the syllables of the TC sentences for the different spectral conditions. Error bars show the standard error.

Figure 2A shows the results of the main experiment in which we assessed the effect of adding silences, spectral content, and measurement (test / retest) on the intelligibility of the TC sentences with a syllabic rate of 15.6 sps. The repeated-measures ANOVA showed a significant main effect of the added silences (i.e., the restoration of the speech rate to 6.1 sps) [F(1, 13) = 52.53, p < 0.00001, ${\eta }_{G}^{2}$ = 0.743] and spectral content [F(1.43, 18.57) = 152.53, p < 0.00001, ${\eta }_{G}^{2}$ = 0.284, Greenhouse–Geisser corrected] on the intelligibility of the TC sentences. There was, however, no main effect of measurement (i.e., comparison of the test and retest data) [F(1, 13) = 3.0, p = 0.107]. Furthermore, there was a significant interaction effect between the adding silences and the spectral content [F(1.71, 22.26) = 12.88, p = 0.0003, ${\eta }_{G}^{2}$ = 0.093, Greenhouse–Geisser corrected]. Post-hoc analyses showed that there was a significant increase in intelligibility when the speech rate was restored to 6.1 sps by adding silences and that for all of the spectral content conditions (p ≤ 0.005). In addition, a richer spectral content resulted in an increase in intelligibility in both the conditions with and without the added silence (p < 0.005).

Figure 2B, shows the benefit of adding silences on intelligibility as a function of spectral content. The benefit of adding silences on intelligibility, when combining both the test and retest measures was 4.97% (SD = 9.45%, range = −12.4 to 34.38%), 16.46% (SD = 14.57%, range = −9.47 to 45.45%), and 23.40% (SD = 18.14%, range = −15.63 to 50.76%) for respectively the 8-channel vocoded, 16-channel vocoded speech, and the speech with the original spectral content. Paired t-tests (based on the RAU scores and combining the test and retest measurements) showed that the benefit in intelligibility was significantly higher in the 16-channel compared to the 8-channel vocoded condition (*t*_*paired*_(27) = −2.48, p = 0.019), and that the original spectral content resulted in a significant higher benefit than the 8-channel (*t*_*paired*_(27) = −3.41, p = 0.002) but not compared to the 16-channel vocoded condition (*t*_*paired*_(27) = −0.37, p = 0.71).

## Discussion

There is ample evidence in the literature that only a limited number of TFS channels are needed to achieve close to perfect intelligibility in quiet purely based on the E modulations in speech^3,5,6,8^. However, these studies all use normal speech rates and hence the modulation spectrum is similar to that encountered in normal-listening situations^42,43^. Furthermore, the rate at which normal-hearing listeners are able to perceive at least 50% of non-vocoded TC speech ranges between 11 to 16 sps^17–19^. The results of the present study show that it is near impossible to perceive TC speech, presented at a rate of 15.6 sps, if only a limited number of TFS channels are available. In addition, we found that the ability to perceive TC speech is associated with the amount of TFS available to the listener. Moreover, we found that the access to TFS results in a larger benefit when the syllabic rate is restored from 15.6 to 6.1 sps by repackaging the syllables with 100 ms of silence (see Fig. 2B). This indicates that there is an interplay between both the E and TFS information available to the listener when listening to TC speech. Therefore, restoring the syllabic rate does not only provide the listener with a better envelope representation but it also enables a better recognition of the speech segments based on its TFS features.

Although the importance of TFS for listening to speech in modulated noise and multi-talker babble is well known^9,11,12,44^, the results of the present study show that TFS is also important for listening to speech in quiet when the E is degraded. In line with our results are those of Meng et al.^18^ who assessed the ability of Mandarin-speaking normal-hearing listeners to perceive vocoded TC speech and cochlear-implant users to perceive non-vocoded TC speech. Their results show a similar reduction in the intelligibility of TC speech when the TFS of the speech signal was degraded. Furthermore, they found that cochlear-implant users, who predominately perceive speech based on E, are less able to perceive TC speech compared to their normal-hearing peers. Similar findings have been reported by Fu et al.^45^.

Ghitza and colleagues^14,25,33^ showed that repackaging TC speech, by inserting silence parts between speech segments, restores intelligibility. The result of the present study shows that this improvement is associated with the amount of TFS available to the listeners. More specifically, the repackaging process does not only restore the E of the speech signal to what is normally encountered in everyday speech, but it also enables a better processing and/or representation of the TFS features of the different speech segments. Several models have postulated that the brain processes speech based on a hierarchical oscillatory mechanism^14,46–49^. The TEMPO model^14,24^, for example, postulates that speech is processed at the cortical level by a cascade of oscillators that operate at different timescales. The theta oscillator (4–10 Hz) synchronizes to the E which drives the beta (6–40 Hz) and gamma (> 60 Hz) oscillator to decode the speech signal at different timescales. Whereas time-compression itself results in a less optimal synchronization of the theta oscillator and consequently the whole cascade of oscillators^14,25^, the results of the present study suggest, within the scope of the TEMPO model, that a reduced TFS affects the decoding process in the beta and gamma oscillator. Nevertheless, the improvement of intelligibility when TC speech is repackaged and how intelligibility of TC and repackaged TC speech depends on the amount of TFS can also be explained with psychoacoustic based auditory processing models. Moore^26^, for example, introduced the spectral-temporal excitation pattern (STEP) model in which the brain is assumed to have internal representations based on the spectral-temporal characteristics of specific sounds. For speech specifically, it is assumed that these STEPs are stored in the long-term memory and that the brain utilizes a ‘warping’ process to map the incoming STEPs to the internal representations, e.g., to account for different speech rates across listeners. One can hypothesize that when the speech rate increases and when in addition the TFS information is limited, matching the incoming STEPs with the internal representations becomes challenging and as a result speech intelligibility decreases.

Although these models provide insight in potential cortical mechanisms that account for the observed results, there is only limited empirical evidence that indicates that this is how the brain processes speech. Recently, Oganian and Chang^50^ found, by means of ECoG, that the superior temporal gyrus (STG) applies a landmark-based temporal analysis. This landmark-based temporal analysis of a continuous speech stream at the level of the STG is based on amplitude-edge detection and results in a discretized representation of the speech stream, consisting of a series of amplitude based events. In case speech is TC, it is possible that the STG cannot encode these edges properly and thereby reduces the discretization of amplitude-based events in speech, i.e., the information which the cortex potentially utilizes to process speech. By introducing silences between the syllables of TC speech this discretization can potentially be restored. In addition, psychophysical research has shown that the ability to perceive short-duration vowels depends on the duration of the silence between sequentially presented short-in-duration vowels^51^. Massaro^51^ found that when the duration of the silences between sequentially presented vowels was shorter than 80–160 ms, vowel discrimination deteriorated. These findings suggest that not only the duration of the speech segment is important for speech perception, but also the processing time available to process each speech segment. In case of TC speech, both the duration of the speech segment and the processing time is altered. One possible explanation is that by inserting the 100 ms silences between syllables, as is the case in the repackaged TC speech, the brain has a longer processing time and hence the interval between speech segments is better matched with the integration times utilized by the auditory system^52,53^. This in turn results in a better intelligibility. Of interest is that the processing time (i.e., the amount of silence needed between sequentially presented sounds) of normal-hearing listeners is shorter for familiar sounds compared to unfamiliar sounds^51^. By changing the amount of spectral channels in vocoding speech, as was done in the present study, not only the amount of TFS is reduced but also the familiarity of the perceived speech segments. This potentially makes it more difficult for the brain to recognize the different segments of the incoming speech stream, which is especially the case for TC speech. The 100 ms silence used in the repackaging TC speech conditions is potentially insufficient to successfully process unfamiliar sounds, such as vocoded speech, compared to familiar sound (e.g., undistorted speech). Alternatively, both the E and the TFS are distorted in such a way, that these effects combined, make that the TC vocoded speech becomes unintelligible. This was especially the case for the eight-channel vocoded TC speech condition.

Although it is not directly clear which processes affect the ability to perceive spectrally degraded TC- and repackaged TC speech, the results of the present study show the E and the TFS are both important for perceiving speech in quiet when presented at fast rates. Furthermore, the findings of the present study provide additional insight in why cochlear-implant users and/or people with a sensorineural hearing impairment struggle to perceive fast speech. They only have limited access to the TFS^18,45^ and therefore predominately rely on the E information to perceive speech and to form their internal representations of the different speech segments throughout development. The latter is especially the case for pre-lingually deaf cochlear-implant users who develop their speech perception based on less than sixteen independent TFS channels^54,55^.

To summarize, we found that the ability to perceive TC speech is affected by the amount of TFS available to the listener. In addition, the benefit listeners experience when TC speech is repackaged, so that the E is restored, is also affected by the amount of TFS in the speech signal. These results show the importance of both the E and TFS when listening in quiet becomes challenging.

## Data Availability

Data is available from the corresponding author upon reasonable request.

## References

[1] Shamma S, Lorenzi C (2013). On the balance of envelope and temporal fine structure in the encoding of speech in the early auditory system. J. Acoust. Soc. Am..

[2] Rosen S (1992). Temporal information in speech: Acoustic, auditory and linguistic aspects. Philos. Trans. R. Soc. Lond. B.

[3] Lorenzi C, Gilbert G, Carn H, Garnier S, Moore BCJ (2006). Speech perception problems of the hearing impaired reflect inability to use temporal fine structure. Proc. Natl. Acad. Sci..

[4] Smith ZM, Delgutte B, Oxenham AJ (2002). Chimaeric sounds reveal dichotomies in auditory perception. Nature.

[5] Drullman R, Festen JM, Plomp R (1994). Effect of reducing slow temporal modulations on speech reception. J. Acoust. Soc. Am..

[6] Shannon RV, Zeng F, Kamath V, Wygonski J, Ekelid M (1995). Speech recognition with primarily temporal cues. Science.

[7] Wouters J, Mcdermott HJ, Francart T (2015). Sound coding in cochlear implants. IEEE Signal Process. Mag..

[8] Zeng F (2005). Speech recognition with amplitude and frequency modulations. Proc. Natl. Acad. Sci..

[9] Gnansia D, Pressnitzer D, Péan V, Meyer B, Lorenzi C (2010). Intelligibility of interrupted and interleaved speech for normal-hearing listeners and cochlear implantees. Hear Res..

[10] Gnansia D, Jourdes V, Lorenzi C (2008). Effect of masker modulation depth on speech masking release. Hear Res..

[11] Nelson PB, Jin S, Carney AE, Nelson DA (2003). Understanding speech in modulated interference: Cochlear implant users and normal-hearing listeners. J. Acoust. Soc. Am..

[12] Qin MK, Oxenham AJ (2004). Effects of simulated cochlear-implant processing on speech reception in fluctuating maskers. J. Acoust. Soc. Am..

[13] Gordon-Salant FS, Friedman PJ, Sarah A (2007). Recognition of time-compressed and natural speech with selective temporal enhancements by young and elderly listeners. J. Speech Lang. Hear. Res.

[14] Ghitza O, Greenberg S (2009). On the possible role of brain rhythms in speech perception : Intelligibility of time-compressed speech with periodic and aperiodic insertions of silence. Phonetica.

[15] Ahissar E (2001). Speech comprehension is correlated with temporal response patterns recorded from auditory cortex. Proc. Natl. Acad. Sci. USA.

[16] Schlueter A, Lemke U, Kollmeier B, Holube I (2014). Intelligibility of time-compressed speech: The effect of uniform versus non-uniform time-compression algorithms. J. Acoust. Soc. Am..

[17] Gransier R, van Wieringen A, Wouters J (2022). The intelligibility of time-compressed speech is correlated with the ability to listen in modulated noise. J. Assoc. Res. Otolaryngol..

[18] Meng Q (2019). Time-compression thresholds for Mandarin sentences in normal-hearing and cochlear implant listeners. Hear Res..

[19] Versfeld NJ, Dreschler WA (2002). The relationship between the intelligibility of time-compressed speech and speech in noise in young and elderly listeners. J. Acoust. Soc. Am..

[20] Carbonell KM (2017). Reliability of individual differences in degraded speech perception. J Acoust Soc Am.

[21] Gordon-Salant S, Friedman SA (2011). Recognition of rapid speech by blind and sighted older adults. J. Speech Lang. Hear. Res..

[22] Wingfield A, Peelle JE, Grossman M (2003). Speech rate and syntactic complexity as multiplicative factors in speech comprehension by young and older adults. Aging Neuropsychol. Cogn..

[23] Ahissar E (2001). Speech comprehension is correlated with temporal response patterns recorded from auditory cortex. Proc. Natl. Acad. Sci..

[24] Ghitza O (2011). Linking speech perception and neurophysiology : Speech decoding guided by cascaded oscillators locked to the input rhythm. Front. Psychol..

[25] Penn LR, Ayasse ND, Wingfield A, Ghitza O (2018). The possible role of brain rhythms in perceiving fast speech: Evidence from adult aging. J. Acoust. Soc. Am..

[26] Moore BCJ (2003). Temporal integration and context effects in hearing. J. Phon..

[27] Ghitza O (2013). The theta-syllable: A unit of speech information defined by cortical function. Front. Psychol..

[28] Lakatos P (2013). The spectrotemporal filter mechanism of auditory selective attention. Neuron.

[29] Teng X, Tian X, Rowland J, Poeppel D (2017). Concurrent temporal channels for auditory processing: Oscillatory neural entrainment reveals segregation of function at different scales. PLoS Biol..

[30] Teng X, Poeppel D (2020). Theta and gamma bands encode acoustic dynamics over wide-ranging timescales. Cereb. Cortex.

[31] van Wieringen A, Wouters J (2008). LIST and LINT : Sentences and numbers for quantifying speech understanding in severely impaired listeners for Flanders and the Netherlands. Int. J. Audiol..

[32] Liu S, Del Rio E, Bradlow AR, Zeng F-G (2004). Clear speech perception in acoustic and electric hearing. J. Acoust. Soc. Am..

[33] Bosker HR, Ghitza O (2018). Entrained theta oscillations guide perception of subsequent speech: Behavioural evidence from rate normalisation. Lang. Cogn. Neurosci..

[34] Moulines E, Charpentier F (1991). Pitch-synchronous waveform processing techniques for text-to-speech synthesis using diphones. Speech Commun..

[35] Boersma, P. & Weenink, D. *Praat: Doing Phonetics by Computer [Computer Program]*. http://www.praat.org (2020).

[36] Van Hirtum T, Moncada-Torres A, Ghesquière P, Wouters J (2019). Speech envelope enhancement instantaneously effaces atypical speech perception in dyslexia. Ear Hear.

[37] Van Hirtum T, Ghesquière P, Wouters J (2021). A bridge over troubled listening: Improving speech-in-noise perception by children with dyslexia. JARO-J. Assoc. Res. Otolaryngol..

[38] The MathWorks Inc. *MatLab 2016B*. (Natick, 2016).

[39] Francart T, van Wieringen A, Wouters J (2008). APEX 3: A multi-purpose test platform for auditory psychophysical experiments. J. Neurosci. Methods.

[40] R Core Team. *R: A Language and Environment for Statistical Computing*. https://www.R-project.org/ (R Foundation for Statistical Computing, 2019).

[41] StudeBaker GA (1985). A ‘rationalized’ arcsine transform. J. Speech Hear Res..

[42] Varnet L, Ortiz-barajas MC, Erra RG, Gervain J, Lorenzi C (2017). A cross-linguistic study of speech modulation spectra. J. Acoust. Soc. Am..

[43] Ding N (2017). Temporal modulations in speech and music. Neurosci. Biobehav. Rev..

[44] Hopkins K, Moore BCJ, Stone MA (2008). Effects of moderate cochlear hearing loss on the ability to benefit from temporal fine structure information in speech. J. Acoust. Soc. Am..

[45] Fu Q-J, Galvin JJ, Wang X (2001). Recognition of time-distorted sentences by normal-hearing and cochlear-implant listeners. J. Acoust. Soc. Am..

[46] Ghitza O (2012). On the role of theta-driven syllabic parsing in decoding speech : Intelligibility of speech with a manipulated modulation spectrum. Front. Psychol..

[47] Poeppel D (2003). The analysis of speech in different temporal integration windows : Cerebral lateralization as ‘asymmetric sampling in time’. Speech Commun..

[48] Giraud A, Poeppel D (2012). Cortical oscillations and speech processing : Emerging computational principles and operations. Nat. Neurosci..

[49] Peelle JE, Davis MH (2012). Neural oscillations carry speech rhythm through to comprehension. Front. Psychol..

[50] Oganian Y, Chang EF (2019). A Speech Envelope Landmark for Syllable Encoding in Human Superior Temporal Gyrus. Sci. Adv..

[51] Massaro DW (1972). Preperceptual images, processingtime, and perceptual units in auditory perception. Psychol. Rev..

[52] Teng X, Tian X, Poeppel D (2016). Testing multi-scale processing in the auditory system. Sci. Rep..

[53] Norman-Haignere SV (2022). Multiscale temporal integration organizes hierarchical computation in human auditory cortex. Nat. Hum. Behav..

[54] Friesen LM (2001). Speech recognition in noise as a function of the number of spectral channels: Comparison of acoustic hearing and cochlear implants. J. Acoust. Soc. Am..

[55] Berg KA (2019). Speech recognition as a function of the number of channels in perimodiolar electrode recipients. J. Acoust. Soc. Am..

